# Supplementary material for: Xenoestrogen concentration in women with endometriosis or leiomyomas: A case-control study
Source: PLoS One. 2024 Jun 4;19(6):e0304766. doi: 10.1371/journal.pone.0304766 (PMC11149880; doi:10.1371/journal.pone.0304766)
Supplement: S1 File — (PDF) [file pone.0304766.s003.pdf]

XENOBEM STUDY-DATABASE ENCODING

E: TYPE OF SURGERY

1. Abdominal Hysterectomy
2. Myomectomy
3. Bilateral Salpingo-oophorectomy
4. Cesarean Section
5. Cystectomy
6. Salpingectomy
7. Appendectomy
8. Unilateral Salpingo-oophorectomy
9. Polypectomy
10. Trachelectomy

F: MEDICAL CONDITION AT THE TIME OF SURGERY

0. No leiomyomas/endometriosis (controls)

1. Leiomyoma

2. Endometriosis

J: PAST MEDICAL HISTORY (PMH) yes/no

K: PAST MEDICAL HISTORY

1. Hypothyroidism
2. Hypertension (HTA)
3. Irritable Bowel Syndrome (IBS)
4. Migraines
5. Other Benign Tumors
6. Polycystic Ovary Syndrome (PCOS)
7. Pelvic Inflammatory Disease (PID)
8. Thyroid Cancer
9. Breast Cancer
10. Epilepsy
11. Hyperthyroidism
12. Type 1 Diabetes (DM1)
13. Others
14. Meningitis
15. Asthma
16. Graves' Disease

17. Juvenile Idiopathic Arthritis
18. Alopecia
19. Pernicious Anemia
20. BRCA
21. Hyperprolactinemia
22. Idiopathic Pancreatitis
23. Muscular Dystrophy
24. Fibromyalgia
25. Arthritis
26. Autoimmune Disease
27. Ulcerative Colitis
28. Pituitary Microadenoma

L: PAST MEDICAL HISTORY SIMPLIFIED

1. Benign Endocrinological Pathology
2. Digestive Pathology
3. Rheumatological Pathology
4. PCOS
5. BRCA
6. Non-gynecological Cancers
7. Respiratory Pathology
8. Neurological Pathology
9. Other Benign Tumors
10. Others

M: SURGICAL HISTORY (SH): yes/no

N: SURGICAL HISTORY

1. Fibroids
2. Endometriosis
3. Breast Cancer
4. Appendectomy
5. Ovarian Cyst
6. Endometrial Polyp
7. Unspecified Hysterectomy
8. Benign Breast Tumor
9. Dilation and Curettage (D&C)
10. Tonsillectomy
11. Cesarean Section
12. Cholecystectomy
13. Adenoidectomy

14. Salpingo-oophorectomy
15. Thyroid Surgery
16. Turbinates
17. Breast Prosthesis
18. Orthopedic
19. Abdominal/Inguinal/Umbilical Hernias
20. Others
21. Herniated Disc
22. Adenomyosis
23. Other Benign Tumors
24. Conization
25. PID

<u>O: SH SIMPLIFIED</u>

1. Fibroids
2. Endometriosis
3. Breast Cancer
4. Benign Ovarian or Uterine Pathology (Cysts, Benign Tumors, Endometrial Polyps, PID, D&C, Cervical Conization)
5. Benign Breast Tumor
6. Digestive System Surgery
7. ENT Surgery (Adenoidectomy, Tonsillectomy, Turbinates)
8. Cesarean Section
9. Thyroid Surgery
10. Breast Prostheses
11. Orthopedic Surgery
12. Others

<u>Q: FAMILY HISTORY</u> of gyn conditions

1. Fibroids
2. Endometriosis
3. Breast Cancer
4. Ovarian Cancer
5. Endometrial Cancer
6. Unspecified Gynecological Cancer

<u>S: HORMONAL CONTRACEPTION</u> (HC) Types

1. Oral Contraceptives
2. Contraceptive patch
3. Vaginal Ring

4. Intrauterine Device (IUD)
5. Contraceptive implant

Y, Z: HORMONE REPLACEMENT THERAPY Y/N, years of use.

AH, AI: RESIDENCE/WORKPLACE (Municipality of residence or work in the last 10 years)

1. Madrid
2. Pozuelo de Alarcón
3. Las Rozas
4. Leganés
5. Tres Cantos
6. Fuenlabrada
7. Getafe
8. Galapagar
9. Guadalajara
10. Alcorcón
11. Alcobendas
12. Alpedrete
13. Colmenar Viejo
14. Alcalá de Henares
15. Móstoles
16. Toledo
17. Cuenca
18. Ciudad Real
19. Villaviciosa de Odón
20. Majadahonda
21. Villanueva
22. Cubas de la Sagra
23. El Escorial
24. Boadilla
25. Parla
26. El Molar
27. Argada
28. Rivas
29. Equatorial Guinea
30. Salamanca
31. Cáceres
32. Palma de Mallorca
33. Mérida
34. Arroyomolinos

35. Jaén
36. Jaca
37. San Sebastián de los Reyes
38. Tomelloso
39. Barcelona
40. Fuerteventura
41. Soria
42. Collado Villalba
43. Valladolid
44. Segovia
45. Tiétar (Cáceres)
46. Jarandilla de la Vera
47. Quintana de la Seseña
48. Hoyo de Manzanares
49. Talavera de la Reina
50. Ávila
51. Villacañas
52. Oviedo
53. Pinto
54. Ibiza
55. Seseña
56. Torrejón
57. Lugo
58. Valmojado (Toledo)
59. Huelva
60. Calamonte
61. Chapinería
62. Venezuela (urban area)
63. Russia (urban area)
64. Venezuela (the Andes)
65. Burgos
66. Venezuela
67. Sant Cugat del Vallès
68. Borox
69. Don Benito
70. Marbella
71. Adeje (Tenerife)

AJ, AK: SIMPLIFIED RECLASSIFICATION OF RESIDENCE/WORKPLACE

1. Madrid Capital
2. Other Municipalities within the Community of Madrid

3. Castilla La Mancha
4. Castilla y León
5. Extremadura
6. Others
