# Supplementary material for: Xenoestrogen concentration in women with endometriosis or leiomyomas: A case-control study
Source: PLoS One. 2024 Jun 4;19(6):e0304766. doi: 10.1371/journal.pone.0304766 (PMC11149880; doi:10.1371/journal.pone.0304766)
Supplement: Attachment — Submitted filename: Reviewers comments.docx [file pone.0304766.s004.docx]

The aim of this study was to examine xenoestrogen concentration differences between women with leiomyomas or endometriosis and control women and to study the relationship between the clinical and sociodemographic characteristics of these patients and their xenoestrogen levels.

Authors reported no association between omental xenoestrogen levels and leiomyomas or endometriosis but their presence, in most participants and their association with highly polluted areas, emphasizes the importance of limiting environmental exposure to these substances. An association between hormonal contraceptive use and xenoestrogen concentration was also found.

I have the following comments for the Authors:

- Please revise your English and check for typos and tense mistakes.
- Methods: Authors should state clearly the outcomes of the study, and which were the related endpoints.
